# miR-206 inhibits osteogenic differentiation of bone marrow mesenchymal stem cells by targetting glutaminase

**DOI:** 10.1042/BSR20181108

**Published:** 2019-03-26

**Authors:** Ying Chen, Yu-Run Yang, Xiao-Liang Fan, Peng Lin, Huan Yang, Xing-Zuo Chen, Xiao-Dong Xu

**Affiliations:** 1 Department of Orthopaedics, China-Japan Friendship Hospital, No.2 Yinghuayuan east street, Beijing 100029, China; 2 Department of Orthopaedics, Hangzhou First People’s Hospital, Nanjing Medical University, Hangzhou 310006, P.R. China

**Keywords:** bone marrow mesenchymal stem cells, glutaminase (GLS), microRNA-206, osteogenic differentiation

## Abstract

Osteoblast-mediated bone formation is a complex process involving various pathways and regulatory factors, including cytokines, growth factors, and hormones. Investigating the regulatory mechanisms behind osteoblast differentiation is important for bone regeneration therapy. miRNAs are known as important regulators, not only in a variety of cellular processes, but also in the pathogenesis of bone diseases. In the present study, we investigated the potential roles of miR-206 during osteoblast differentiation. We report that miR-206 expression was significantly down-regulated in human bone marrow mesenchymal stem cells (BMSCs) at days 7 and 14 during osteogenic induction. Furthermore, miR-206 overexpressing BMSCs showed attenuated alkaline phosphatase (ALP) activity, Alizarin Red staining, and osteocalcin secretion. The mRNA levels of osteogenic markers, Runx2 and Osteopontin (OPN), were significantly down-regulated in miR-206 overexpressing BMSCs. We observed that significantly increased glutamine uptake at days 7 and 14 during the osteogenic induction and inhibition of glutamine metabolism by knocking down glutaminase (GLS)-suppressed osteogenic differentiation of BMSCs. Here, we discover that miR-206 could directly bind to the 3′-UTR region of *GLS* mRNA, resulting in suppressed GLS expression and glutamine metabolism. Finally, restoration of GLS in miR-206 overexpressing BMSCs led to recovery of glutamine metabolism and osteogenic differentiation. In summary, these results reveal a new insight into the mechanisms of the miR-206-mediated osteogenesis through regulating glutamine metabolism. Our study may contribute to the development of therapeutic agents against bone diseases.

## Introduction

miRNAs are non-coding, single-stranded, and short (22–24 nts) RNAs that post-transcriptionally regulate the gene expression by binding to the 3′-UTR of their target mRNAs [1]. miRNAs play important roles in a variety of cellular processes including proliferation, migration, differentiation, and apoptosis [2]. Moreover, miRNAs are also involved in the diverse pathology of human bone diseases [3,4].

Bone is the main element of the skeletal system [5]. Osteoblast-mediated bone formation is a complex but finely orchestrated process [6]. One of the important regulators of bone formation is the bone morphogenetic protein (BMP), which plays major roles during the osteoblast lineage-specific differentiation, and later, in bone formation [7]. Bone marrow mesenchymal stem cells (BMSCs) are the progenitor cells for osteoblasts, adipocytes, and chondrocytes [8]. The potential of BMSCs to differentiate into osteoblasts or adipocytes can be utilized to repair and reconstruct bone tissue injury or disease [9], providing a potential strategy for clinical treatments. The osteogenic differentiation of BMSCs involves a complex regulatory network. However, the detailed molecular mechanisms that regulate the differentiation of BMSCs remain unclear.

Multiple miRNAs that regulate osteogenesis or adipogenesis have been characterized [10]. Amongst them, miR-188 was highly expressed in aged mice and humans, leading to regulate the differentiation of BMSCs into osteoblasts [11]. In addition, Runx2, an osteogenic marker, was known as a target of miR-204 and miR-211 [12]. BMP2, which controls the switch between bone and muscle differentiation has been reported to be targetted by multiple miRNAs [13]. Recent study revealed that miR-206 was down-regulated in osteoblast cells during osteogenesis [14], suggesting that miR-206 has regulatory functions during the differentiation of BMSCs. Despite these findings, the functions of miR-206 during the differentiation and lineage commitment of BMSC needs further studies. Here, we found that overexpression of miR-206 inhibits osteogenic differentiation of BMSCs. Moreover, miR-206 suppresses glutamine metabolism, which is essential for differentiation of BMSCs. The putative target of miR-206 in glutamine metabolism during the osteogenic differentiation of BMSCs will be investigated.

## Materials and methods

### BMSC culture and osteogenic induction

BMSCs were obtained from the American Type Culture Collection (ATCC) (Manassas, VA, U.S.A.) and cultured in growth medium (Dulbecco’s modified Eagle’s medium (DMEM) supplemented with 10% FBS, 2 mM of l-glutamine, and 1× penicillin/streptomycin) or osteogenic induction medium. The osteogenic induction medium was DMEM supplemented with 10% FBS, 2 mM of l-glutamine, 1× penicillin/streptomycin, 100 nM of dexamethasone, 10 mM of β-glycerophosphate, and 50 μM of l-ascorbic acid-2-phosphate (Sigma, St. Louis, MO, U.S.A.). Cells were cultured in a cell incubator at 37°C and 5% CO_2_.

### Alkaline phosphatase activity assay and Alizarin Red staining

The alkaline phosphatase (ALP) activity assays were performed using an enzymatic colorimetric ALP kit (Roche Diagnostics, Minneapolis, MN, U.S.A.) according to the previous report [15]. The Alizarin Red staining was performed as previously described [15]. The Alizarin red staining was quantitated by spectrophotometry at 540 nm. All experiments were repeated three times and performed in triplicate.

### Osteocalcin secretion assay

The osteocalcin secretion was measured using the Human Osteocalcin ELISA Kit (ab195214) (Abcam, Cambridge, U.K.) according to the manufacturer’s instructions. Briefly, BMSCs were grown in osteogenic induction medium as described above in six-well plates at 2.5 × 10^6^ cells per well for 7 days. At day 4, cells were transfected with negative control or miR-206 precursor or miR-206 precursor plus glutaminase (GLS) overexpression vector, for 3 days. The osteocalcin secretion was measured at day 7 and normalized by cell number of each experimental group.

### miRNA and siRNA transfection

miR-206 precursors and their negative controls were purchased from Ribobio (Guangzhou, China), and were transfected into BMSCs at 50–60% confluence at 50 nM concentration with Lipofectamine 3000 (Invitrogen, Carlsbad, CA, U.S.A.). Negative-control siRNA and siGLS were purchased from Sigma (St. Louis, MO, U.S.A.) and were transfected into BMSCs at 50 nM concentration with Lipofectamine 3000 (Invitrogen, Carlsbad, CA, U.S.A.). After 48 h, cells were harvested for miRNA, protein, or mRNA analysis.

### Glutamine uptake

The glutamine uptake assay was performed using the Glutamine Detection Assay Kit (ab197011) from Abcam (Cambridge, U.K.) according to the manufacturer’s instructions. The relative glutamine uptake was normalized by cell numbers of each experimental group.

### Quantitative reverse transcription PCR analysis

Total RNA was extracted by TRIzol reagent (Invitrogen) and the integrity of RNA was examined by A_260_/A_280_ ratio using NanoDrop (Thermo Fisher Scientific, Waltham, MA, U.S.A.). cDNA synthesis was performed using the iScript™ cDNA Synthesis Kit (Bio-Rad, Hercules, CA, U.S.A.). The reaction conditions were: 25°C for 5 min, 46°C for 20 min, and 95°C for 1 min. Primer sequences used for the present study were: RUNX2: Forward: 5′-GCACCAAGTCCTTTTAATCCACAA-3′, Reverse: 5′-GGGAAGACTGTGCCTGCCT-3′; Osteopontin (OPN): Forward: 5′-ACTCGAACGACTCTGATGATGT-3′, Reverse: 5′-GTCAGGTCTGCGAAACTTCTTA-3′; GAPDH: Forward: 5′-GGATTTGGTCGTATTGGG-3′, Reverse: 5′-GGAAGATGGTGATGGGATT-3′. Real-time PCR was performed with SYBR Green reagents (Bio-Rad, Hercules, CA, U.S.A.) according to the manufacturer’s instructions. GAPDH was used as an internal control for mRNA detection, RNU6 was used as an internal control for miRNA detection. The relative mRNA expression was calculated by 2^−ΔΔ*C*^_t_ method.

### Luciferase reporter assay

The wild-type or mutant 3′-UTR of the *GLS* mRNA were amplified and cloned into pMIR-Report luciferase vector by inserting between the SacI/HindIII sites. GLS 3′-UTRs were mutated using the Quickchange II site-directed mutagenesis kit (Stratagene). Cells were co-transfected with negative control miRNA or miR-206 precursor at 25 or 50 nM and WT or mutant pMIR-REPORT-Luc DNA construct for 48 h. Luciferase assay was performed using a dual luciferase reporter assay system (Promega) using a luminometer. Luminescence reading was normalized with the *Renilla* luciferase value. Experiments were performed in triplicate and all experiments were repeated three times.

### Western blot analysis

The GLS expressions were detected by Western blot analysis. After treatments, cells were harvested and lysed by RIPA buffer containing protease inhibitor cocktail (Sigma, St. Louis, MO, U.S.A.). Lysates were incubated at 4°C for 30 min followed by centrifugation at 12000 rpm for 10 min at 4°C. The protein concentrations were measured by Bradford assay (Beyotime, China). Protein samples (30 μg) were separated by 10% SDS/PAGE. Proteins were transferred on to 0.22 μm-pore-diameter nitrocellulose filter (NC) membranes (Millipore, U.S.A.). Membranes were blocked with 5% BSA for 1 h at room temperature, followed by incubation with primary rabbit antibodies against GLS (ab93434, Abcam, U.S.A.) (1:1000) and β-actin (Boster Bio, China) (1:2000) for overnight at 4°C. After washing with TBS/T three times, the membranes were incubated with secondary antibody (1:3000) for 1 h at room temperature. Then, the membranes were washed and scanned by using an Odyssey Infrared Imaging System (LI-COR, U.S.A.).

### Statistical analyses

Results are presented as the mean ± S.D. Comparisons between experimental groups were analyzed using Prism 6.0 (GraphPad Software Inc., San Diego, CA, U.S.A.). The differences between two groups were calculated by Student’s *t* test. Comparisons of multiple groups were calculated using one-way ANOVA. All experiments were repeated three times. *P*<0.05 was considered statistically significant.

## Results

### miR-206 is down-regulated during osteogenic differentiation

**Figure 1:**
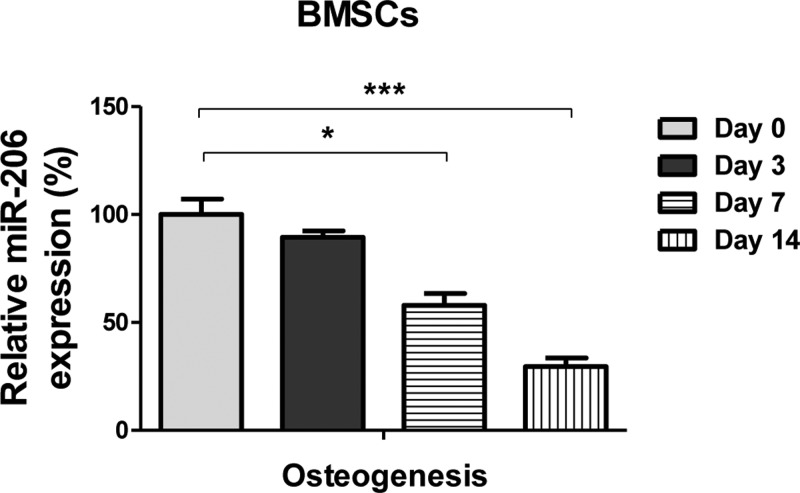
miR-206 is down-regulated in BMSCs during osteogenic differentiation qRT-PCR was performed to analyze the expressions of miR-206 in BMSCs under osteogenic induction conditions at days 0, 3, 7, or 14. Data are shown as the mean ± S.D. *P<0.05; ***P<0.001. Abbreviation: qRT-PCR, quantitative reverse transcription PCR.

Previous findings revealed that osteogenic differentiation of BMSCs is post-transcriptionally regulated by miRNAs [14]. Specifically, miR-206 is expressed in osteoblasts and its expression decreases during osteoblast differentiation [14]. To understand whether miR-206 is involved in osteogenic differentiation of BMSCs, we investigated the pattern of miR-206 expression during the osteogenic differentiation. As we expected, the expressions of miR-206 were significantly down-regulated in BMSCs at days 7 and 14 during osteogenic induction (Figure 1), coinciding with the previous reports, that the miR-23a/b was significantly down-regulated in BMSCs during the ageing process, leading to regulate the osteoblast differentiation [16]. These data suggest a suppressive role of miR-206 during the osteogenic differentiation of BMSCs.

### Overexpression of miR-206 inhibits the osteogenic differentiation of BMSCs

**Figure 2:**
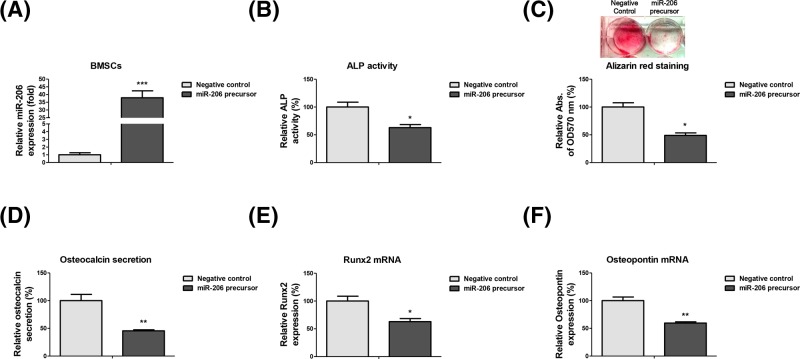
miR-23a/b inhibits the osteogenic differentiation of BMSCs (**A**) BMSCs were transfected with native control or miR-206 precursor for 48 h. Expressions of miR-206 were measured by qRT-PCR. RNU6 was the internal control. (**B**) BMSCs were transfected with native control or miR-206 precursor for 48 h, the relative ALP activity. (**C**) Alizarin red staining and (**D**) Osteocalcin secretion were analyzed. (**E**) BMSCs were transfected with native control or miR-206 precursor for 48 h. The mRNA expressions of Runx2 and (**F**) OPN were detected by qRT-PCR. GAPDH was the internal control. Data are shown as the mean ± S.D. *P<0.05; **P<0.01; ***P<0.001. Abbreviation: qRT-PCR, quantitative reverse transcription PCR.

We next investigated whether overexpression of miR-206 could regulate the osteogenic differentiation. MiR-206 precursor or their negative control was transfected into BMSCs for functional investigation (Figure 2A). Cells were subsequently induced to osteogenic differentiation. Expectedly, under osteogenic induction, miR-206 overexpressing BMSCs showed attenuated ALP activity (Figure 2B), Alizarin Red staining (Figure 2C), and osteocalcin secretion (Figure 2D). These three osteoblast differentiation markers consistently demonstrated that overexpression of miR-206 could suppress osteogenic differentiation of BMSCs. Accordingly, the mRNA levels of osteogenic markers, Runx2 (Figure 2E) and OPN (Figure 2F) were significantly down-regulated in miR-206 overexpressing BMSCs. Taken together, the above results demonstrated a suppressive role of miR-206 during the osteogenic differentiation of BMSCs.

### Glutamine metabolism is essential for osteogenic differentiation of BMSCs

**Figure 3:**
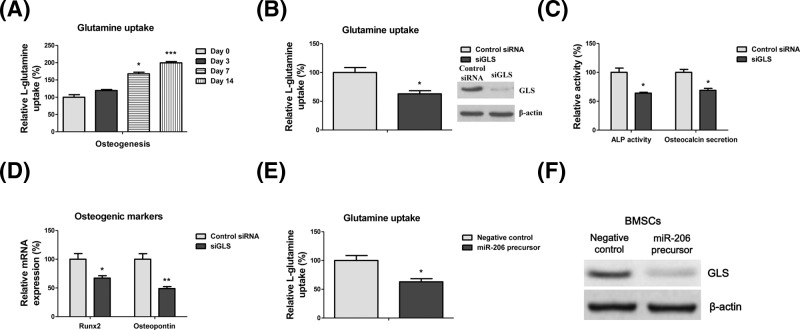
Glutamine metabolism is essential for osteogenic differentiation of BMSCs (**A**) BMSCs were treated with osteogenic induction condition for 0, 3, 7, or 14 days, the relative glutamine uptakes were measured. (**B**) BMSCs were transfected with control siRNA or siGLS for 48 h. The glutamine uptake (**C**) ALP activity and Osteocalcin secretion were measured. (**D**) BMSCs were transfected with control siRNA or siGLS for 48 h. The mRNA expressions of Runx2 and OPN were detected by qRT-PCR. GAPDH was the internal control. (**E**) BMSCs were transfected with negative control or miR-206 precursor for 48 h. The relative glutamine uptake and (**F**) GLS protein expression were measured. β-actin was the internal control. Data are shown as the mean ± S.D. *P<0.05; ***P<0.001

Recent study revealed an increase in glutamine catabolism during bone formation [17], suggesting that the glutamine metabolism is essential for osteogenic differentiation. To assess the mechanism behind the miR-206-modulated osteogenic differentiation, we assessed the glutamine uptake. Expectedly, glutamine uptake was significantly increased at days 7 and 14 during osteogenic induction (Figure 3A). It was known that the GLS deaminates glutamine to form glutamate, the rate-limiting step in glutamine metabolism [18]. We next knocked down GLS expression by specific siRNA and found that glutamine uptake was significantly decreased (Figure 3B), indicating GLS as a target protein for regulating *in vitro* osteogenesis of BMSCs. With attenuated GLS expression, BMSCs showed down-regulated ALP activity and osteocalcin secretion (Figure 3C). Moreover, the mRNAs of Runx2 and OPN were down-regulated in GLS knocked-down BMSCs (Figure 3D). We then hypothesized that miR-206 regulates glutamine metabolism. To test this, we assessed the glutamine uptake and GLS protein expressions in BMSCs with or without miR-206. Results showed that overexpression of miR-206 significantly suppressed glutamine uptake and down-regulated the GLS expressions (Figure 3E,F). Taken together, the above results demonstrated that miR-206 regulates glutamine metabolism, which is essential for the osteogenic differentiation of BMSCs.

### miR-206 directly targets GLS

**Figure 4:**
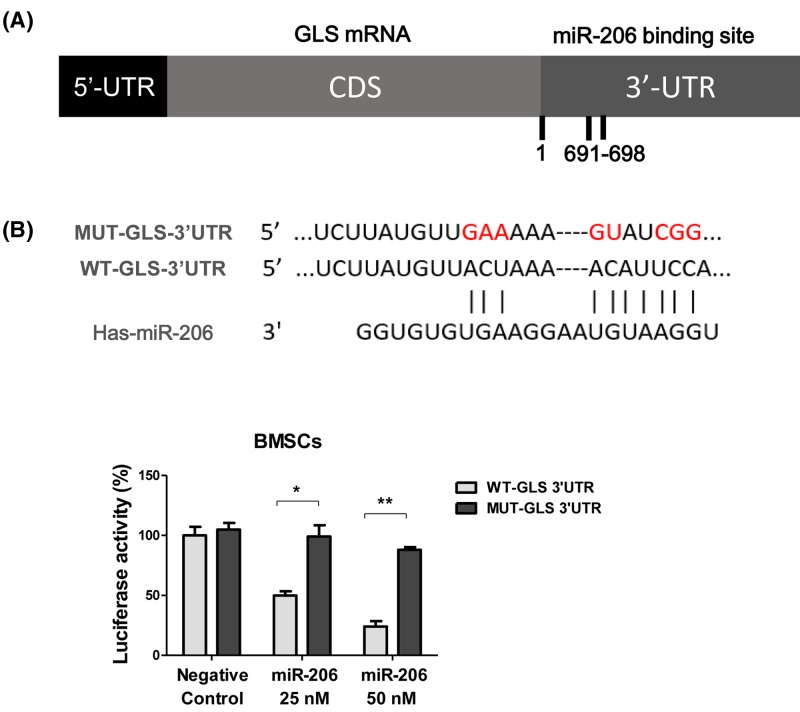
miR-206 directly binds to the 3′-UTR region of GLS (**A**) The predicted miR-206 binding sites on 3′-UTR region of GLS. (**B**) The mutant construct of the binding site (upper). BMSCs were co-transfected with control miR or miR-206 at 25 or 50 nM and wild-type or mutant GLS 3′-UTR for 48 h. The relative luciferase activities were measured (lower). Data are shown as the mean ± S.D. *P<0.05; **P<0.01.

It was known that miRNAs regulated the expression of their target mRNAs by binding to the coding sequences or the 3′-UTRs [1,2]. To access whether miR-206 could directly target the key enzymes that regulate glutamine metabolism, we first used TargetScan (http://www.targetscan.org/vert_72/) to predict the potential target genes of miR-206. Amongst the multiple potential target genes predicted, we found the GLS, which had been reported to participate in the regulation of glutamine metabolism through catalyzing the conversion of glutamine into glutamate [17]. Sequence analysis showed one miR-206 binding site at the position 691–698 nts of 3′-UTR of GLS (Figure 4A). Since we have observed that miR-206 suppressed GLS protein expression (Figure 3F), to clarify whether miR-206 could directly target the GLS gene, a luciferase reporter construct including the predicted binding site of the GLS 3′-UTR (WT-GLS-3′UTR) and multiple mutant nucleotides of the binding sites, was generated (Figure 4A). We co-transfected WT-GLS or MUT-GLS along with negative control miRNAs or miR-206 (25, 50 nM) into BMSCs and assessed the gradient effects of miR-206 on luciferase translation by luciferase enzyme activity. As expected, transfection with miR-206 was able to repress the luciferase activity of the GLS 3′-UTR reporter gene, whereas MUT-GLS prevented this suppression (Figure 4B), confirming that miR-206 particularly binds to the predicted 3′-UTR region of GLS.

### Restoration of GLS results in recovery of glutamine metabolism and osteogenic differentiation

**Figure 5:**
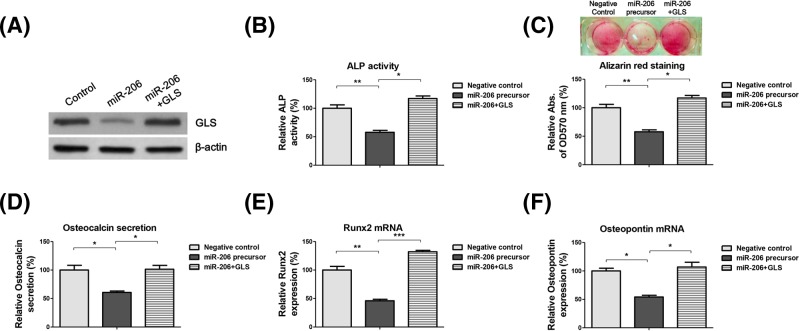
Restoration of GLS recovers glutamine metabolism and osteogenic differentiation of BMSCs (**A**) BMSCs were transfected with control vector or miR-206 precursor or miR-206 plus GLS for 48 h. The expressions of GLS were detected by Western blot. β-actin was the internal control. (**B**) BMSCs were transfected with control vector or miR-206 precursor or miR-206 plus GLS for 48 h. The relative ALP activity, (**C**) Alizarin Red staining, and (**D**) Osteocalcin secretion were analyzed. (**E**) BMSCs were transfected with control vector or miR-206 precursor or miR-206 plus GLS for 48 h. The mRNA expressions of Runx2 and (**F**) OPN were detected by qRT-PCR. GAPDH was the internal control. Data are shown as the mean ± S.D. *P<0.05; **P<0.01; ***P<0.001. Abbreviation: qRT-PCR, quantitative reverse transcription PCR.

The above results demonstrated the correlation between miR-206-mediated glutamine metabolism and osteogenic differentiation of BMSCs. To determine whether miR-206 directly regulates osteogenic differentiation through targetting GLS, we transfected BMSCs with control vector, miR-206 precursor alone, or co-transfected GLS overexpression vector with miR-206 precursor (Figure 5A). Results from Western blot showed that co-transfection of GLS and miR-206 successfully rescued GLS protein expression (Figure 5A). We then measured ALP activity (Figure 5B), Alizarin Red staining (Figure 5C), and osteocalcin secretion (Figure 5D). These three markers of osteoblast differentiation consistently demonstrated that restoration of GLS in miR-206 overexpressing BMSCs rescued osteogenic differentiation potential. Moreover, we found that restoration of GLS recovered the mRNA expression of osteogenic markers, Runx2 (Figure 5E) and OPN (Figure 5F). Taken together, these results confirmed that the miR-206-regulated osteogenic differentiation of BMSCs was indeed through GLS targetting.

## Discussion

The osteoblast-mediated bone formation, a complex yet finely regulated process, is important for maintaining bone homeostasis [5,6]. Differentiation of mature osteoblasts from BMSCs was regulated by complex signaling pathways, transcription factors, and cytokines [8,19]. BMSCs are progenitor cells with potential to differentiate into osteoblasts or adipocytes [19], providing a strategy for clinical treatments of various bone diseases and injuries. However, understanding the regulatory mechanism behind the differentiation of BMSCs requires further investigation. Recently, multiple studies revealed critical roles of miRNAs in regulating osteogenic or adipogenic differentiation. It has been described that miR-23a/b was prominently down-regulated in BMSCs of aged mice and humans [16]. Overexpression of miR-23a/b also promoted the osteogenic differentiation of BMSCs [16]. In addition, miR-15b was reported to promote osteoblast differentiation of hMSCs by suppressing SMAD7 [20]. MiR-20a also promotes osteoblast differentiation from hMSC by targetting peroxisome proliferator-activated receptor (PPAR) [21]. In this study, we identified miR-206 as a negative regulator for the osteogenic differentiation of BMSCs. Overexpression of miR-206 significantly suppressed *in vitro* osteogenic differentiation of BMSCs by down-regulating osteogenesis markers, illustrating that miR-206 has the potential for the development of therapeutic agent for bone disease. Yet, the detailed molecular pathway, upstream of miR-206 suppression, during osteogenic induction is still under investigation. It was known that during the osteogenic induction, the TGF-β/BMP pathway gets activated [22]. In addition, studies demonstrated that TGF-β treatment inhibits miR-206 expression during the myogenic differentiation [23], suggesting that the up-regulated TGF-β pathway inhibits miR-206 expressions, resulting in the GLS up-regulation during osteogenic induction.

Recent studies demonstrated that miRNAs have the potential to become therapeutic agents for the bone tissue regeneration [24]. Transplanting stem cells with manipulated miRNAs could enhance the differentiation of MSCs toward chondrogenic or osteogenic lineages [24]. Research from Chen et al. [25] transfected MSCs with pre-miR-34a, anti-miR-34a, and control miRNAs, and loaded them on to hydroxyapatite/tricalcium phosphate (HA/TCP) scaffolds, and then implanted them subcutaneously into immunocompromised mice. They reported that hMSCs transfected with anti-miR-34a resulted in more than 3.5-fold increase in bone formation [25]. Thus, miRNAs contribute to direct the transplanted stem cells toward the desired cell fate. Moreover, early deliveries of miRNAs help to prevent the onset and progression of bone disease by inducing the regeneration of skeletal tissues [26].

It was known that glutamine as well as other essential nutrition metabolism are interrelated and involved in BMSC differentiation [27]. Osteoblast differentiation is associated with elevated metabolic requirements, which are provided by glucose, fatty acids, lactate, or glutamine [28]. Glutamine is converted into glutamate and then into α-ketoglutarate, which is necessary for bone formation [17]. In the present study, we observed that overexpression of miR-206 suppressed the glutamine metabolism during osteogenic differentiation of BMSCs, presenting miR-206 a potential therapeutic target for bone disease. We identified GLS as a direct target of miR-206 in BMSCs. Furthermore, rescue experiments with restoration of GLS determined that the miR-206-mediated BMSC differentiation was through direct targetting of GLS. In clinical application, it is possible to transfect antisense miR-206 into hMSCs using Lipofectamine, followed by the local delivery of these cells on to a HA/TCP scaffold to regenerate bone tissues [29]. Although our *in vitro* study using BMSCs has limitation, we are now focussing on the construction of an *in vivo* bone formation model. In summary, these results suggest that miR-206 has a critical role in BMSC differentiation.

## Abbreviations

ALP: alkaline phosphatase
BMP: bone morphogenetic protein
BMSC: bone marrow mesenchymal stem cell
DMEM: Dulbecco’s modified Eagle’s medium
GLS: glutaminase
HA/TCP: hydroxyapatite/tricalcium phosphate
hMSC: human mesenchymal stem cell
OPN: osteopontin
TGF-β: transforming growth factor β
